# Supplementary material for: The paralogues MAGOH and MAGOHB are oncogenic factors in high-grade gliomas and safeguard the splicing of cell division and cell cycle genes
Source: RNA Biol. 2023 Jun 9;20(1):311–22. doi: 10.1080/15476286.2023.2221511 (PMC10259345; doi:10.1080/15476286.2023.2221511)
Supplement: Supplemental Material [file KRNB_A_2221511_SM2481.zip › Barreiro.et-al.SupplementaryData.pdf]

SUPPLEMENTAL MATERIAL

The paralogues MAGOH and MAGOHB are oncogenic factors in high grade gliomas and safeguard the splicing of cell division and cell cycle genes

Rodrigo Barreiro[1,2,#], Gabriela D. A. Guardia[1,#], Fabiana M. Meliso[1,#], Xiufen Lei[2,3], Wei-Qin Li[1,5], Wei-Qui Li' staff, Andre Savio[3], Helena B. Conceição[1], Rafael L. V. Mercuri[1], Martin Fellermeyer[3], Tesha Landry[3], Mei Qiao[3], Lorea Blazquez[6,7], Jernej Ule[6,7], Luiz O. F. Penalva[3,8,*], Pedro A. F. Galante[1,*]

1 - Hospital Sirio-Libanes, São Paulo, 01308-060, Brazil.

2 - Departamento de Bioquimica, Instituto de Química - Universidade de São Paulo, São Paulo 05508-000, Brazil.

3 - Greehey Children's Cancer Research Institute, University of Texas Health Science Center at San Antonio, San Antonio, TX 78229, USA.

4 - Department of Nutritional Sciences, Dell Pediatric Research Institute, Dell Medical School, The University of Texas at Austin, Austin, TX, 78712, USA.

5 - Shanghai Changzheng Hospital, Second Military Medical University, Shanghai, China.

6 - The Francis Crick Institute, 1 Midland Road, London, NW1 1AT, UK. jernej.ule@ki.si.

7 - UK Dementia Research Institute, King's College London, London, UK. jernej.ule@ki.si.

8 - Department of Cell Systems & Anatomy, University of Texas Health Science Center at San Antonio, San Antonio, TX 78229, USA.

# These authors contributed equally.

* Corresponding authors: Luiz O. F Penalva and Pedro A F Galante

A)

```
MAGOH       ------ATGGAGAGTGACTTTTATCTGCGTTACTACGTGGGGCACAAGGGCAAGTTCGGC      54
MAGOHB      ATGGCTGTGGCTAGCGATTTCTACCTGCGCTACTACGTAGGGCACAAGGGCAAGTTTGGG      60
                   ***  ** ** ** ** ***** ******** ***************** **

MAGOH       CACGAGTTCCTGGAGTTTGAGTTTCGACCGGACGGGAAGTTAAGATATGCCAACAACAGC      114
MAGOHB      CACGAGTTTCTGGAGTTCGAATTTCGGCCGGACGGAAAGCTTAGATATGCCAACAACAGC      120
            ******** ******** ** ***** ******** *** * ******************

MAGOH       AATTACAAGAATGATGTCATGATCAGAAAAGAGGCTTATGTACATAAAAGCGTGATGGAG      174
MAGOHB      AATTACAAAAATGATGTCATGATCAGAAAAGAGGCTTATGTGCACAAGAGTGTAATGGAA      180
            ******** ******************************** ** ** ** ** *****

MAGOH       GAACTGAAGAGAATAATTGACGACAGTGAAATTACCAAAGAGGATGATGCATTGTGGCCT      234
MAGOHB      GAACTGAAGAGAATTATTGATGACAGTGAAATTACAAAAGAAGATGATGCTTTGTGGCCT      240
            ************** ***** ************** ***** ******** *********

MAGOH       CCTCCTGACCGAGTGGGCCGGCAGGAGCTTGAAATCGTCATTGGAGATGAACACATTTCT      294
MAGOHB      CCCCCTGATAGGGTTGGCCGACAGGAGCTTGAAATTGTAATTGGAGATGAGCACATATCT      300
            ** *****  * ** ***** ************** ** *********** ***** ***

MAGOH       TTTACAACATCAAAAATTGGTTCCCTTATTGATGTCAATCAATCCAAGGATCCAGAAGGC      354
MAGOHB      TTTACCACATCAAAAATAGGTTCTCTTATTGATGTAAATCAGTCAAAGGATCCTGAAGGC      360
            ***** *********** ***** *********** ***** ** ******** ******

MAGOH       TTACGAGTATTTTATTATCTTGTCCAGGACCTGAAGTGTTTGGTCTTCAGTCTTATTGGA      414
MAGOHB      CTTCGAGTATTTTACTATTTGGTACAAGACTTGAAATGTTTAGTTTTCAGTCTTATTGGA      420
             * *********** *** * ** ** *** **** ***** ** ***************

MAGOH       TTACACTTCAAGATTAAACCAATCTAG 441
MAGOHB      TTACACTTCAAGATTAAACCAATTTAA 447
            *********************** **
```

B)

```
MAGOH       --MESDFYLRYYVGHKGKFGHEFLEFEFRPDGKLRYANNSNYKNDVMIRKEAYVHKSVME      58
MAGOHB      MAVASDFYLRYYVGHKGKFGHEFLEFEFRPDGKLRYANNSNYKNDVMIRKEAYVHKSVME      60
              : ********************************************************

MAGOH       ELKRIIDDSEITKEDDALWPPPDRVGRQELEIVIGDEHISFTTSKIGSLIDVNQSKDPEG      118
MAGOHB      ELKRIIDDSEITKEDDALWPPPDRVGRQELEIVIGDEHISFTTSKIGSLIDVNQSKDPEG      120
            ************************************************************

MAGOH       LRVFYYLVQDLKCLVFSLIGLHFKIKPI        146
MAGOHB      LRVFYYLVQDLKCLVFSLIGLHFKIKPI        148
            ****************************
```

Supplementary Figure 1. CLUSTAL Omega (1.2.4) multiple sequence alignment. Nucleotide (A) and protein (B) sequence alignment of MAGOH and MAGOHB CDS region. Asterisk (*) in the third line represents a perfect match between both sequences. Colon (:) represents a match of aminoacids of the same class.

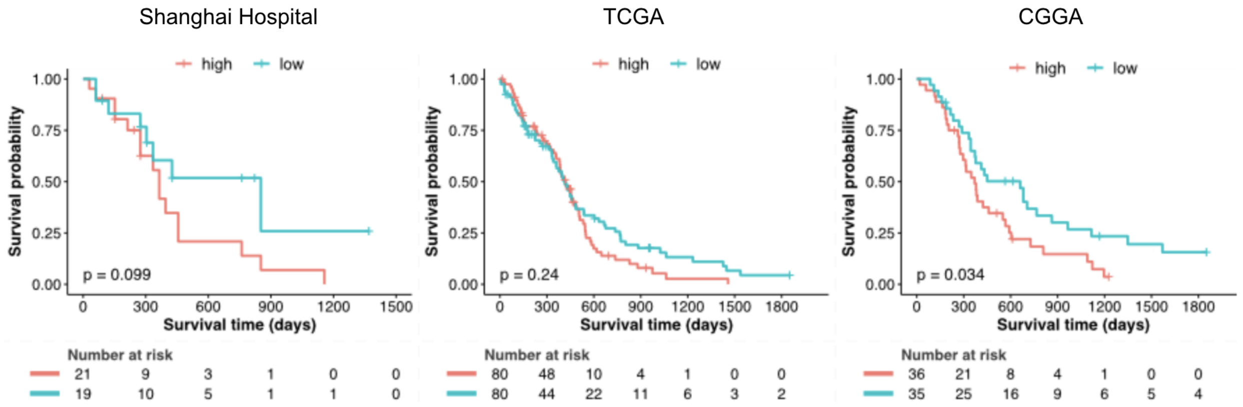

Supplementary Figure 2. Survival curves between high grade glioma patients with high (red) and low (blue) combined MAGOH+MAGOHB expression in three independent cohorts.

A)

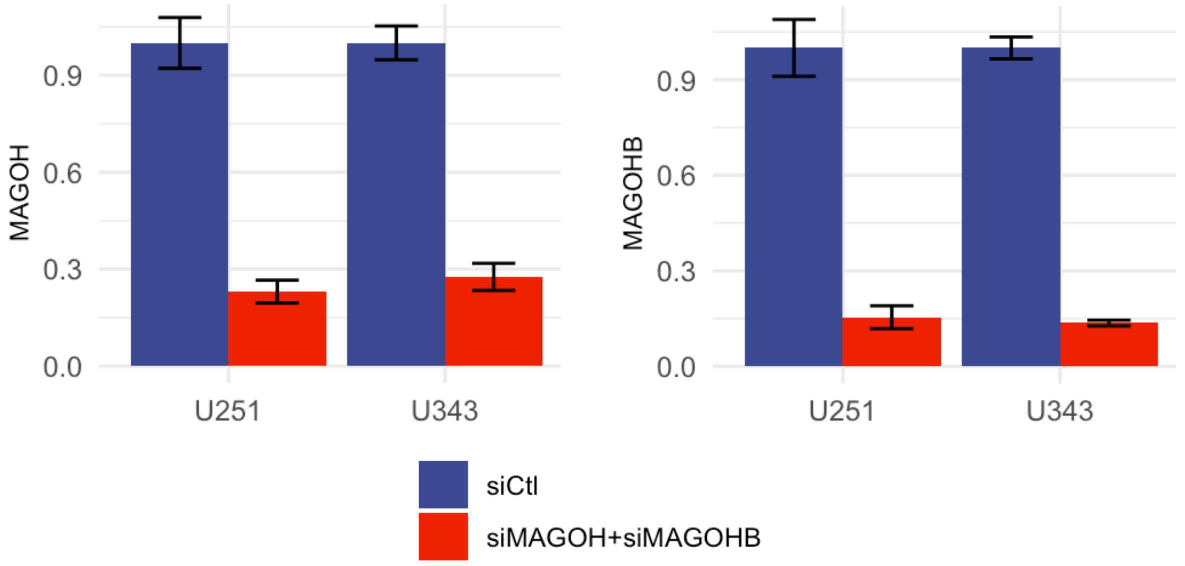

B)

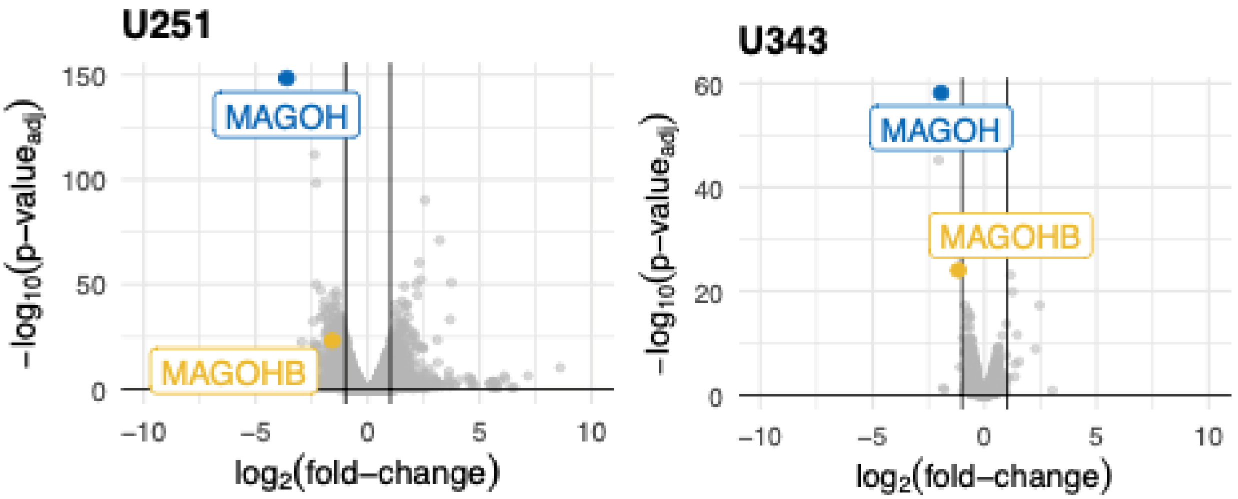

Supplementary Figure 3. MAGOH and MAGOHB knockdown essays. (A) Real Time PCR for MAGOH/MAGOHB in U251 and U343 transfected with control siRNA (siCtl) or MAGOH/MAGOHB small interfering RNA (siRNA: siMAGOH+siMAGOHB). (B) Volcano plot of differentially expressed genes in MAGOH and MAGOHB knockdown essays in glioma stem cells U251 and U343.

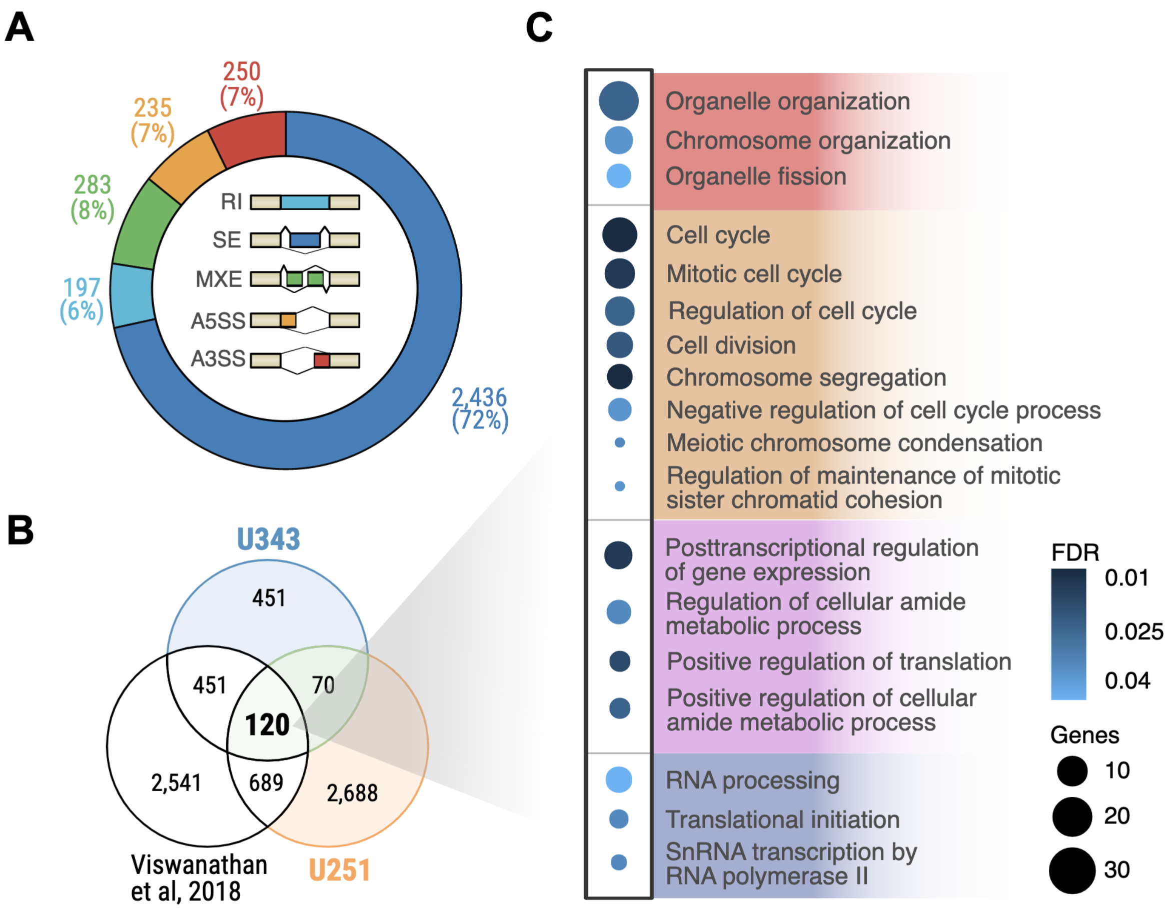

Supplementary Figure 4. Alternative splicing events found in our cell lines (U251 and U343) and into external data (Viswanathan et al 2018). A) rMATS alternative splicing event classification on Viswanathan MAGOH/MAGOHB knockdown data: Skipped exons (SE), retained introns (RI), mutually exclusive exons (MXE), and alternative 5′ and 3′ splice sites (A5SS and A3SS). B) Overlapping alternative splicing events between the three assays. C) Gene Ontology's Biological Processes enrichment for the genes with alternative splicing in all three assays.

A)

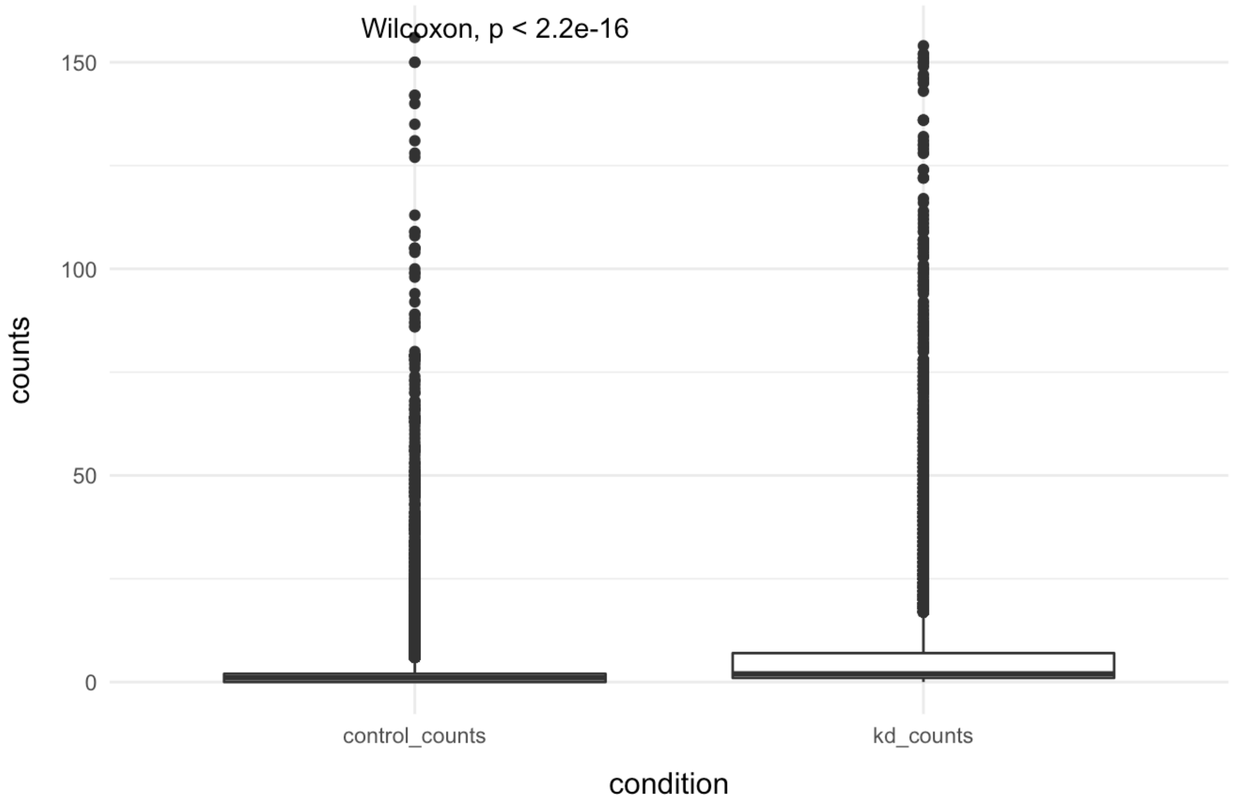

B)

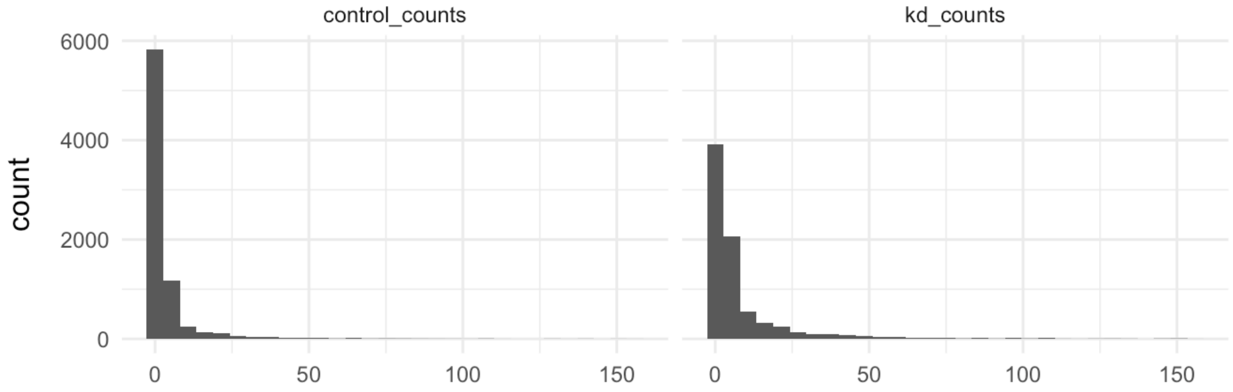

Supplementary Figure 5. Number of reads supporting exon skipping events in U251 and U343 wild type (transfected with siCtrl: control) and with MAGOH/B knockdown (KD).

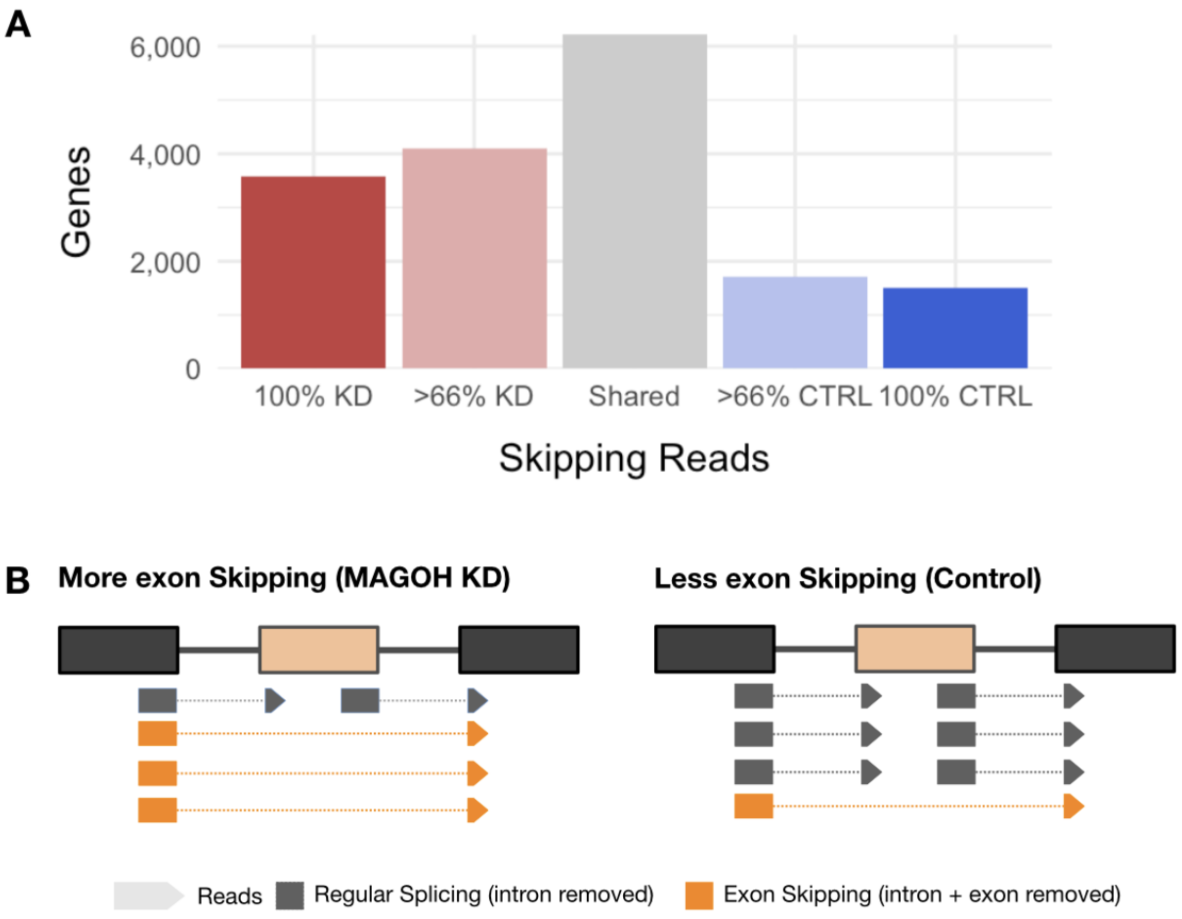

Supplementary Figure 6. MAGOH/B KD cells present more alternative splicing than control cells (CTRL). A) Number of exon skipping events found only in KD cells (red), preferentially in KD cells (light red; where more than 66% of reads are from KD cells), only in control cells (blue), or preferentially in control cells (light blue). Shared events (reads from both KD and control cells) are represented by the gray bar. B) MAGOH and MAGOHB knockdown cell (KD) lines exhibit more exon skipping events than control (CTRL) cells.

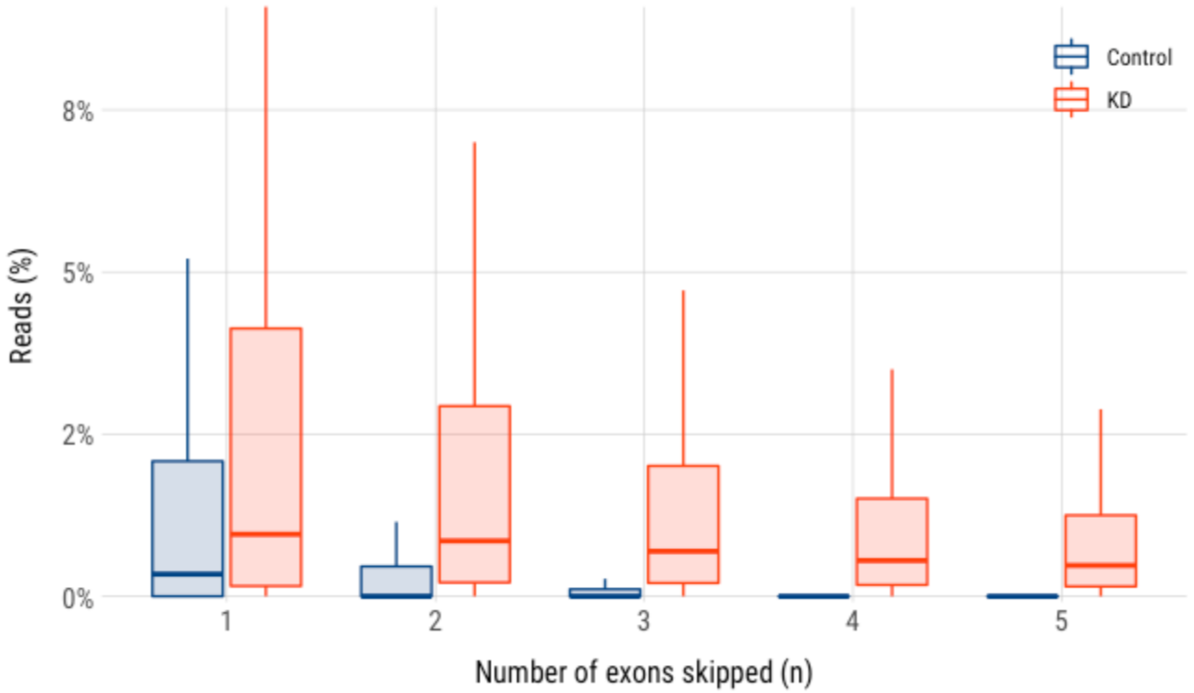

Supplementary Figure 7. Box plot showing percentage of reads supporting exon skipping of 1 to 5 exons in U251 and U343 wild type (transfected with siCtrl: Control) and with MAGOH/B knockdown (KD).
